# Acting fast to prevent post-partum deaths

**DOI:** 10.2471/BLT.23.020923

**Published:** 2023-09-01

## Abstract

A new approach to treating post-partum haemorrhage is having a substantial impact on maternal deaths. Tatum Anderson reports.

It is the mothers lost to post-partum haemorrhage (PPH) that weigh on Dr Hadiza Shehu Galadanci’s mind. Professor of obstetrics and gynaecology at the Aminu Kano Teaching Hospital in Kano State, Nigeria, Galadanci is often called in to help mothers, who – having just delivered their baby – are suddenly faced with the prospect of dying.

“The birth of a child should be a happy time for the mother, but when the bleeding starts everything can change very rapidly,” she says. “The mother is very frightened and sometimes, despite our best efforts, she slips from our hands.”

Commonly defined as blood loss of 500 ml or more within 24 hours after birth, PPH can be caused by a lack of uterine contracture after delivery, retained placenta, an inverted or ruptured uterus, and cervical, vaginal, or perineal tears.

Often life-threatening, PPH is also common. “Of the 134 million births worldwide each year, around 14 million are complicated by post-partum haemorrhage, with most of the women affected living in lower-income countries,” says Dr Ioannis Gallos, a Medical Officer working in the World Health Organization’s (WHO) Sexual and Reproductive Health and Research department.

According to Gallos, PPH is the leading cause of maternal mortality, taking an estimated 70 000 lives every year, equating to roughly 1 in 4 of the 287 000 maternal deaths globally.

The overwhelming majority of these deaths occur in low- and middle-income countries, Nigeria alone accounting for an estimated 20 000. Women who survive can be left with life-long reproductive disability and trauma.

“[PPH is] all the more tragic because it is preventable.”

Ioannis Gallos

“It’s all the more tragic because it is preventable,” says Gallos, listing interventions that include uterine massage after delivery of the placenta, the administration of oxytocin or misoprostol (both of which induce uterine contraction), or tranexamic acid (a blood-clotting agent).

Skilled birth attendants also have the option of inserting a uterine balloon tamponade into the uterus and inflating it to compress blood vessels. As a last resort, surgical removal of the uterus may be needed to save the mother’s life.​

While not all these interventions are always available, many are and, according to Gallos, it is the way they are implemented that gives rise to problems.

Galadanci concurs. “Broadly speaking, we’re too slow in responding,” she says, noting that three main reasons have been identified as the cause of the delays.

The first is the failure to recognize PPH early enough. “More than half of PPH cases go unnoticed,” Galadanci says. “Health workers see the blood, but they underestimate the amount being lost.”

The second cause for delay is the lack of preparation of the medical products needed or the paperwork required to obtain those products. “In many cases prescriptions or other paperwork needs to be signed by a doctor,” Galadanci says. “When medical products are not available on-site, patients’ relatives are asked to go to an outside pharmacy to find them.”

The third main cause of delay is the wait-and-see approach typically taken to treatment. “We administer one drug, and then wait to see whether it is effective or not,” says Galadanci. “In the meantime, the woman is bleeding and before you realise it, she's in serious trouble, especially if there is limited access to services such as blood transfusion or surgery.”

To address these gaps in response, researchers at the University of Birmingham in the United Kingdom of Great Britain and Northern Ireland, working in collaboration with WHO, and researchers in Kenya, Nigeria, South Africa and United Republic of Tanzania have developed a new protocol designed to accelerate PPH response times.

The main aim of the protocol, known as E-MOTIVE, is to help reduce maternal deaths in line with sustainable development goal target 3.1 – a global Maternal Mortality Ratio (MMR) of less than 70 maternal deaths per 100 000 live births by 2030.

“After improving for many years, maternal mortality trends have flattened over the last five,” explains Gallos, who headed up research on E-MOTIVE at Birmingham University prior to moving to WHO.

A report published by WHO and partners in February 2023 (*Trends in maternal mortality 2000 to 2020*) showed that global MMR decreased only slightly between 2015 and 2020, from 227 to 223 deaths per 100 000 live births.

“E-MOTIVE is designed to change this picture,” Gallos says.

E-MOTIVE directly addresses the three main causes for delay. First, by ensuring that staff are ready to spring into action as soon as a problem is identified. Key to achieving readiness is the preparation of a cart or case which contains all the medical products required.

To address the problem of late diagnosis, birth attendants are trained to use a plastic blood-collection drape that goes under the woman giving birth, and includes a calibrated pouch which gives an accurate indication of the amount of blood being lost.

Finally, to avoid delays in treatment, the protocol calls for simultaneous administration of indicated treatments, comprising uterine massage, oxytocic drugs, tranexamic acid, intravenous fluids, immediate examination and escalation of treatment where necessary.

“The core proposal from WHO was to bundle key interventions together without waiting to see what happens next,” Gallos explains.

The E-MOTIVE protocol was tested in 80 hospitals across Kenya, Nigeria, South Africa and the United Republic of Tanzania over a period of seven months between 2021 and 2022, in a randomized controlled trial that covered over 210 000 births. The trial was funded by the Bill and Melinda Gates Foundation.

The results were surprising. “We expected to see a 25% drop in severe postpartum haemorrhage (defined as blood loss greater than 1000 ml) using the protocol but ended up seeing a 60% drop,” says Arri Coomarasamy, Professor of Gynaecology and Reproductive Medicine at Birmingham University, and head of the E-MOTIVE research team. According to Coomarasamy, some of the hospitals in the trial went on to achieve an 80% reduction in severe PPH.

WHO is now working on guidance that will account for all aspects of E-MOTIVE implementation including cost-effectiveness. In the meantime, an implementation trial is being conducted in Pakistan, to verify that the protocol works in countries outside the African Region.

There is evidence that the protocol may also be of benefit in upper middle-income countries, as indicated by a 14-hospital study carried out across the Western Cape, Eastern Cape and KwaZulu-Natal in South Africa.

“After implementation [of the protocol] there were zero deaths.”

Hadiza Shehu Galadanci

According to Professor Sue Fawcus, at the Department of Obstetrics and Gynaecology at the University of Cape Town, who oversaw the study there, death from PPH is the second most common cause of maternal death in South Africa, but the most preventable, with 90% of deaths being clearly preventable through better care supported by a properly functioning health system.

She observed multiple benefits from the protocol, including a reduction in the requirement for blood transfusions. “We observed an apparent reduction in PPH in the postnatal ward, where there are fewer staff and more patients, because cases are being detected while mums are still in the labour ward, enabling earlier intervention,” she explains.

Another benefit was the obvious impact on midwife empowerment. “As midwives, we can immediately implement the bundle and don’t have to wait for a doctor to decide what to do,” says Sister Thea Williams, Operational Manager of the Maternity Ward at Vredenburg Provincial Hospital in the Western Cape, adding that doctors are not always available in the maternity ward where she works. “Being able to take decisions and act on them gives you confidence as a midwife, and the patient feels safer with you too,” she says.

However, Williams also points to a number of challenges which will need to be addressed as the protocol is rolled out. Training is one. As nurse champion on the E-MOTIVE project in her hospital, Williams was in charge of overseeing training, and reports that doctors and nurses were so busy on shift, there was no chance to train them in how to use the new bundle and the drapes unless they came in on their days off.

Fawcus highlights similar concerns, noting the need for constant training. “There’s turnover of staff, agency staff, new students. So, we were continually trying to keep people acquainted with the intervention,” she says, adding that the situation will improve when the E-MOTIVE approach becomes national policy backed by widespread teaching.

Implementation will also need to address the issue of blood-collection drapes, which are single-use items and not biodegradable. In South Africa, the drapes are also imported, raising supply chain concerns. The Bill & Melinda Gates Foundation has agreed to supply drapes until a long-term sustainable solution is found.

Such teething problems aside, there is considerable excitement about the new protocol, based largely on the clear impact it is having. In Nigeria, having seen the difference it makes, Professor Galadanci’s staff are anxiously waiting to see E-MOTIVE fully implemented in all maternity hospitals in Nigeria. “In one of the 19 intervention hospitals in the E-MOTIVE trial, 25 women died of PPH between January and June 2022 prior to implementation,” says Galadanci. “In the six months after implementation, there were zero deaths. Zero. I mean, wow.”

**Figure:**
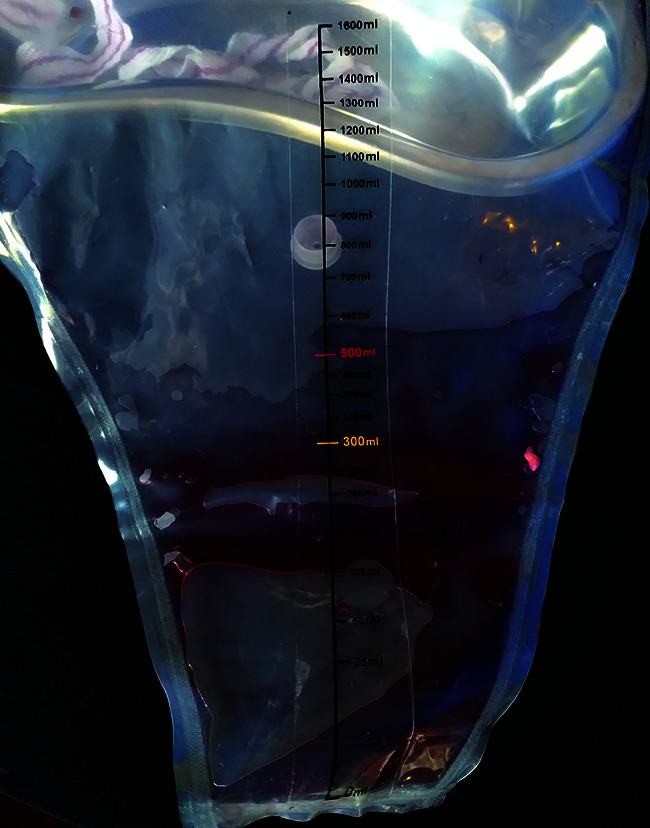
Blood-collection drape calibrated pouch

Arri Coomarasamy /Birmingham University

**Figure:**
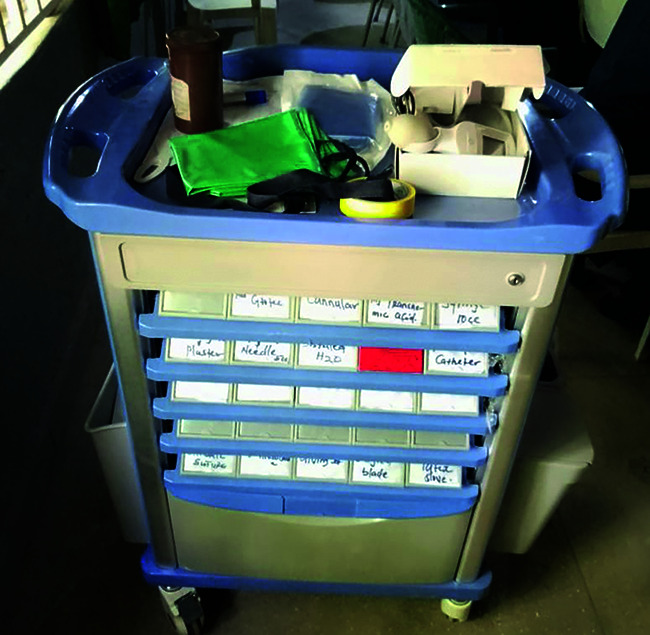
E-MOTIVE quick response cart

Arri Coomarasamy /Birmingham University

